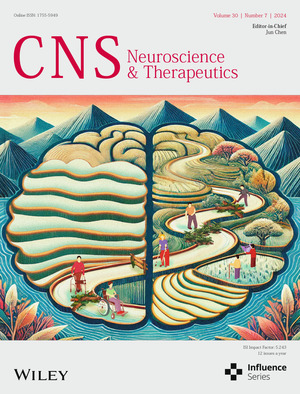# Additional Cover

**DOI:** 10.1111/cns.14909

**Published:** 2024-08-02

## Abstract

The cover image is based on the article *Exploring Functional and Structural Connectivity Disruptions in Spinocerebellar Ataxia Type 3: Insights from Gradient Analysis* by Xingang Wang et al., https://doi.org/10.1111/cns.14842.